# Primary blast causes mild, moderate, severe and lethal TBI with increasing blast overpressures: Experimental rat injury model

**DOI:** 10.1038/srep26992

**Published:** 2016-06-07

**Authors:** Vikas Mishra, Maciej Skotak, Heather Schuetz, Abi Heller, James Haorah, Namas Chandra

**Affiliations:** 1 Center for Injury Biomechanics, Materials and Medicine (CIBM3), Department of Biomedical Engineering, New Jersey Institute of Technology, Newark, NJ 07102-1982, USA; 2 Department of Pharmacology and Experimental Neuroscience, University of Nebraska Medical Center, Omaha, 68198, NE,USA

## Abstract

Injury severity in blast induced Traumatic Brain Injury (bTBI) increases with blast overpressure (BOP) and impulse in dose-dependent manner. Pure primary blast waves were simulated in compressed gas shock-tubes in discrete increments. Present work demonstrates 24 hour survival of rats in 0–450 kPa (0–800 Pa∙s impulse) range at 10 discrete levels (60, 100, 130, 160, 190, 230, 250, 290, 350 and 420 kPa) and determines the mortality rate as a non-linear function of BOP. Using logistic regression model, predicted mortality rate (PMR) function was calculated, and used to establish TBI severities. We determined a BOP of 145 kPa as upper mild TBI threshold (5% PMR). Also we determined 146–220 kPa and 221–290 kPa levels as moderate and severe TBI based on 35%, and 70% PMR, respectively, while BOP above 290 kPa is lethal. Since there are no standards for animal bTBI injury severity, these thresholds need further refinements using histopathology, immunohistochemistry and behavior. Further, we specifically investigated mild TBI range (0–145 kPa) using physiological (heart rate), pathological (lung injury), immuno-histochemical (oxidative/nitrosative and blood-brain barrier markers) as well as blood borne biomarkers. With these additional data, we conclude that mild bTBI occurs in rats when the BOP is in the range of 85–145 kPa.

Exposure to blasts is one of the leading causes of trauma experienced by military personnel as a result of widespread use of high explosives. Our unique animal models of primary blast waves generated in compressed gas shock tubes in discrete increments shows that 24-hour survival of animals depends on the magnitude of blast overpressure (BOP)^1^,^2^. Blast-induced traumatic brain injuries (bTBI) are classified as primary, secondary, tertiary, and quaternary^3^,^4^,^5^, based on the type of biomechanical loading. An incident pressure of a shockwave on the body within the time duration of few-to-ten milliseconds causes primary blast injury, while secondary blast injuries are caused by impact of high-velocity fragmentation and debris. Tertiary blast injuries result when the body is violently accelerated and is forced to impact other objects. Quaternary blast injuries are caused by exposure to heat and toxic gases released resulting from explosive detonation^6^. The present study is focused on primary bTBI that was recognized as a separate neuropathological condition during the World War I and dubbed as ‘shell shock’^7^. Soldiers afflicted with shell shock in the battle field sustained plethora of neurological deficits (or even death) without any visible injuries long after the shelling had ended^8^.

The emergence of bTBI among active military personnel in recent Iraq and Afghanistan war gained considerable attention and remains an important public health problem^5^,^9^,^10^. In the past decade, the Department of Defense reported more than 200,000 head injuries due to combat-related incidents and in non-deployed environment^11^. The severity of brain injury is clinically classified as mild^12^,^13^,^14^, moderate^15^,^16^,^17^,^18^, severe^3^,^19^,^20^,^21^,^22^,^23^, and vegetative state TBI^3^ as per 15-point Glascow Coma Scale (GCS) in humans (including blast TBI cases^24^,^25^,^26^). Over 150,000 of these head injured military personnel were diagnosed with mild Traumatic Brain Injury (mTBI) and Post-Traumatic Stress Disorder (PTSD) exhibiting a wide range of neurological and psychological symptoms^4^,^5^. Blast mTBI is the most prevalent form of trauma among deployed military populations and it is labeled as the invisible signature wound among combat troops^27^,^28^. Currently, mTBI is clinically diagnosed using six categories of symptoms: 1) alteration and 2) loss of consciousness, 3) post-traumatic amnesia, 4) Glasgow Coma Scale (score of 13–15, 30 min. after the injury), 5) focal neurological signs and 6) brain imaging^29^.

To study the origin of bTBI, live-fire testing, compressed-gas shock tubes, combustion shock tubes and small explosion shock tubes have been used with small animals (e.g. rats and mice) and large animals (e.g. swine). A number of shock-tube models of mice^30^,^31^,^32^ and rats^1^,^33^,^34^,^35^,^36^,^37^,^38^ have been developed to represent ‘mild-moderate-severe’ injury scale using a single^23^,^33^,^35^,^36^,^37^,^39^,^40^,^41^,^42^ or a set of blast intensities^15^,^31^,^38^,^43^,^44^. Despite enormous gain of knowledge and advancement of bTBI research in animal model, there is lack of consensus and information about the rationale for selection of range of blast overpressure and impulse as accepted predictors of bTBI. These models are further confounded by variations in the test specimen locations such as inside^1^,^35^ versus at the end/outside^33^,^38^,^45^ of the shock tube, pressure data recording location, secondary loading as a consequence of inappropriate specimen-to-shock tube cross section ratio, lack of proper head restraint, and use of table-top devices^46^,^47^. Though each of these experiments show evidence of effect of blasts on biological materials, in order to study the effect of primary blast within the range of practical relevance (strengths of explosives and stand-off distances responsible for mTBI in military personnel), it is necessary to generate pure shock pulses with 0–450 kPa range and duration between 2 ms and 8 ms^1^,^35^,^48^. Thus, our objective in this study is to identify the thresholds of injury scale and in specific, to determine the mTBI range based on predicted mortality rate (PMR). The reason we have chosen to focus on mTBI is because more than 80% of bTBI cases belong to this category, while the general methodology will be applicable to all other ranges^46^,^47^.

In the present studies, we exposed the animals to BOP ranging from 60 kPa to 450 kPa (equal to 8.7–65.3 psi) with duration of 2–8 milliseconds, as this corresponds to the shock-time pulse at a stand-off distance of 2–10 meters away from 1–100 kg of C4 explosion^49^. A sigmoidal survival-BOP dose-response curve was obtained which was then used to delineate the upper threshold of mild TBI based on the logistic regression model. We then carefully examined this range using other potential indicators of injury. This range was found to compromise the integrity of the blood-brain barrier (BBB). The rationale is based on our recent demonstration of BBB oxidative injury and other studies, that BBB damage is one of the major and most frequently investigated mechanisms of traumatic brain injury in blast TBI^50^,^51^,^52^.

## Results

### Determination of predicted mortality rate (PMR)

**Figure 1:**
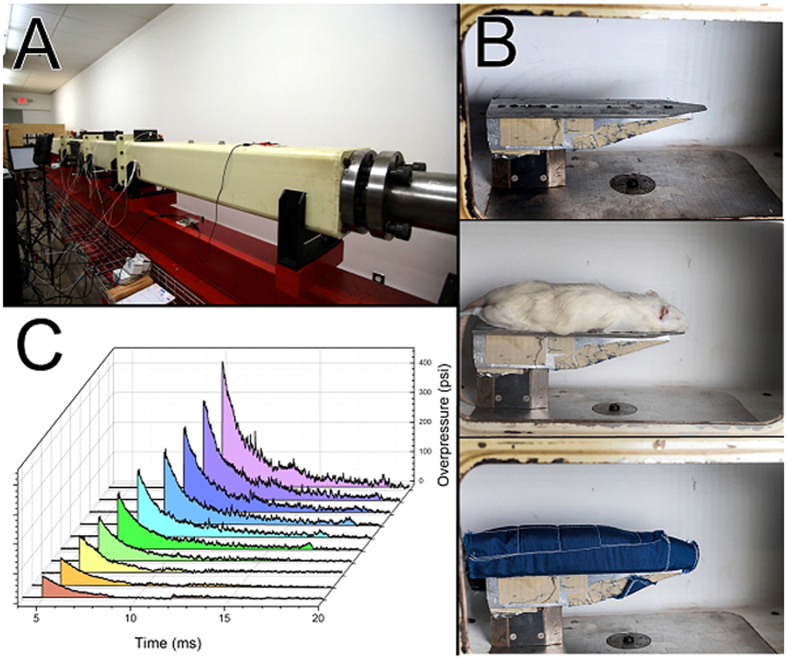
(**A**) An overview of the 9-inch square cross section, 6 meters long shock tube instrumented with pressure sensors. (**B**) Aerodynamic rat holder mounted in the test section (top), with rat placed on top (middle) and animal wrapped in a harness to minimize head and body motion during blast exposure (bottom). (**C**) Representative overpressure profiles (C) as measured in the test section at the location of the animal’s head for 10 experimental groups used in our study.

**Figure 2:**
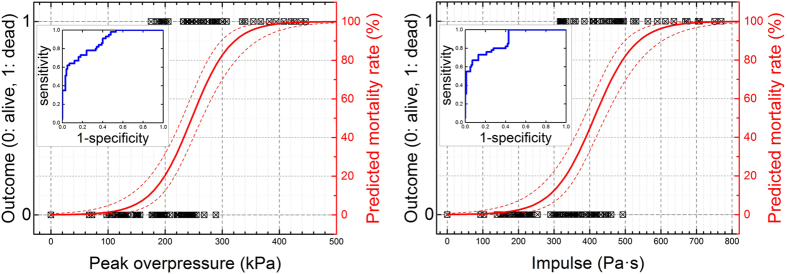
The logistic regression dose-response model for rats exposed to single blast with intensity in the range of 60–450 kPa peak overpressure (left) and corresponding impulse (90–780 Pa∙s, right). The insets present receiver operating characteristic (ROC) curves for respective logistic regression fits with areas under ROC curves: 0.878 and 0.882. McFadden R^2^ = 0.389 and 0.412, for fits using peak overpressure and impulse as independent variables, respectively. Dashed lines indicate confidence intervals of the model.

All the test animals were in a prone position with a head restrained (Fig. 1B), and thus no artifacts associated with uncontrolled head acceleration were present in this bTBI model, as demonstrated elsewhere^1^. The net movement of the specimen did not exceed 3 mm as determined by analysis of captured high speed video. We exposed 13 rats per discrete incident peak overpressure to develop a dose-response linear regression model for predicted mortality rate (PMR) as a function of BOP and corresponding impulse values (Fig. 2). We did not observe any mortality of animals exposed to blast intensity lower than 170 kPa (Fig. 2, left) or impulse 300 Pa·s (Fig. 2, right). However mortality rate gradually increased from 170 kPa to 300 kPa, and mortality rate was 100% above 300 kPa (or 500 Pa·s impulse). These accounted for an immediate loss (not delayed deaths, i.e. animals were removed from the shock tube in less than a minute after the shock wave exposure) of 45 rats as a consequence of shock wave exposure, consistent with our previous work^1^.

The insets in Fig. 2 present receiver operating characteristic (ROC) curves for respective logistic regression fits with the following areas under ROC curves: 0.878 (left) and 0.882 (right), for BOP and impulse, respectively. Analysis from these models indicated good predictive power of mortality rate using peak overpressure and impulse as metrics to gauge injury risk under primary blast. The McFadden pseudo-R^2^ values of 0.389 and 0.412 were obtained for fits using peak overpressure and impulse as independent variables, respectively. These values indicate a highly satisfactory quality of fit.

### Primary blast impacts mild pulmonary injury

**Figure 3:**
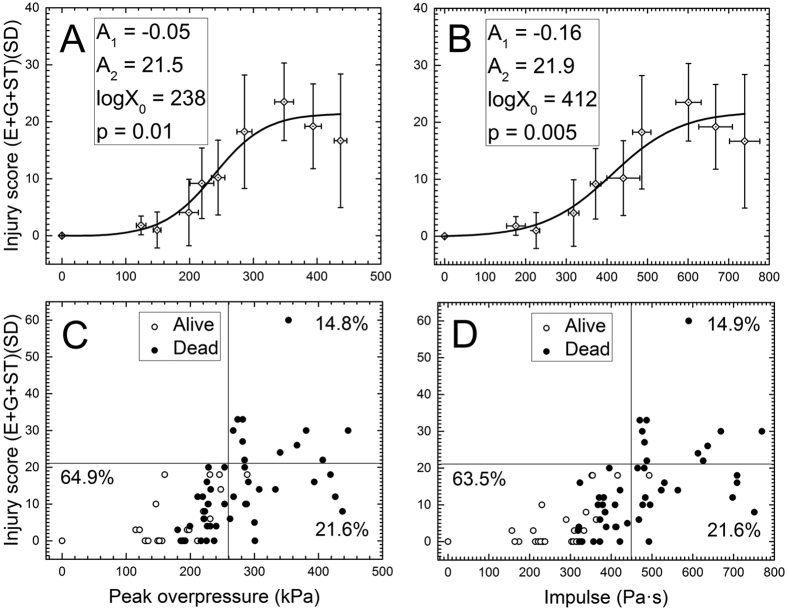
Lung injury scores for rats exposed to a single blast. The dose-response model was used to fit the IS as a function of peak overpressure (**A,C**) and impulse (**B,D**). The scattergrams (**C,D**) illustrate individual scores and their distribution among the cohort of 75 rats evaluated in this test. The value of 21 is the upper limit of the slight lung injury level as defined by Yelverton^53^. The vertical lines (peak overpressure of 260 kPa (**C**) or impulse of 450 Pa·s (**D**)) correspond to 50% predicted mortality rate according to the dose-response model in Fig. 2. There are six animals which died after the blast and had no apparent lung injuries (score of zero).

Animals tested in prone position have their abdomen partially protected from the blast wave by the aluminum holder (Fig. 1B) used in our experiments^1^. The observed levels of pulmonary injury expressed using Yelveton’s scoring system^53^ revealed a low level of injury (Fig. 3A,B). However, we observed an increasing trend of injury score with increasing peak overpressure and impulse. We observed only a few cases where pathological score exceeded 21 for the blast strength higher than 300 kPa BOP with high standard deviations (Fig. 3C). A score of 21 is considered as a cut-off threshold for mild pulmonary injury^53^. The pathological score at 50% PMR (at 260 kPa) was found to be less than 10, while the score was less than 4 in the 60–190 kPa range. Moreover, there are six animals which died as a result of blast exposure and had no lung injury (score of zero, Fig. 3C,D). These results suggested minimal pulmonary injuries and thus, we conclude lung injury is not a viable indicator of PMR.

### Blast induced bradycardia

**Figure 4:**
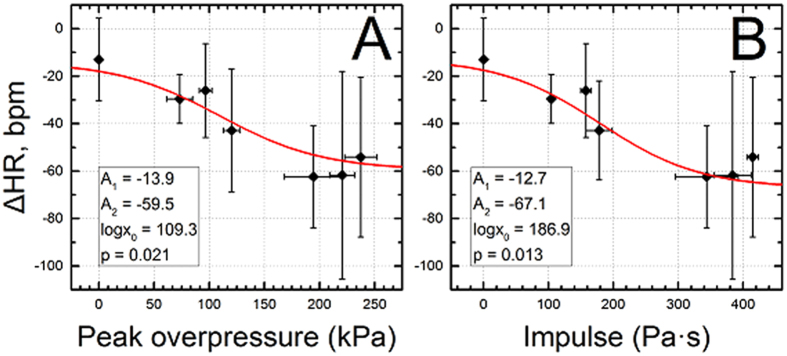
Blast induced bradycardia as a function of peak overpressure (**A**) and impulse (**B**). In both cases, the dose-response function (1) was used to model the pathological response and resulting parameters are listed in respective insets in both figures. All blast exposed groups have statistically significant heart rate decrease versus control (p < 0.05).

We evaluated the functional changes in the heart rate, blood oxygen saturation (spO_2_) and perfusion index in the 60–250 kPa peak overpressure range over period of 30 minutes before and after blast exposure. We found that the onset of bradycardia occurred immediately after the blast exposure even at 60 kPa (Fig. 4, p = 0.01, power: 0.85 vs control). The values of the differential average heart rates (ΔHR) decreased gradually with increase in blast intensity: −29 ± 10 (60 kPa), −26 ± 20 (100 kPa), −43 ± 26 (130 kPa), −62 ± 21 (190 kPa), −62 ± 43 (230 kPa) and −62 ± 24 (250 kPa) bpm. These data were modeled using a simple dose-response function to quantify the characteristics of ΔHR as a function of two blast parameters, the peak overpressure and the impulse. This mathematical modeling generated asymptotic values of A_1_ and A_2_: −13.9 and −59.5 (for peak overpressure, Fig. 4A), and −12.7 and −67.1 (for impulse, Fig. 4B), respectively. The calculated inflection points (log(x_0_)) are 109.3 kPa and 186.9 Pa·s for both blast parameters (Fig. 4). The control group was not correlated with any of the blast exposure groups (p < 0.05).

### Induction of oxidative/nitrosative stress markers

**Figure 5:**
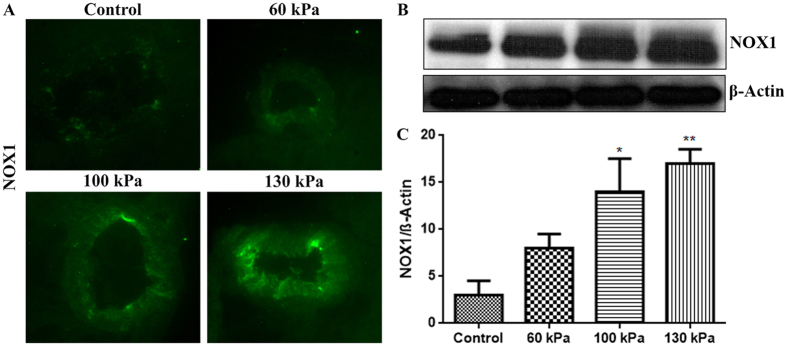
Mild TBI range of blast-wave exposure induces NADPH oxidase expression in rat brain microvessels. (**A**) A representative of immunofluorescent staining of NOX1 in intact microvessels of brain cross sections from rats subjected to a single exposure to 60, 100, or 130 kPa peak overpressure, and control. (**B**) Corresponding Western blot of NOX1 and housekeeping protein, β-actin. (**C**) Bar graphs show the quantitative results of the NOX1 immunoreactive fluorescence intensities. Values are mean ± SEM (n = 4) with p-value ≤0.01 compared with control.

**Figure 6:**
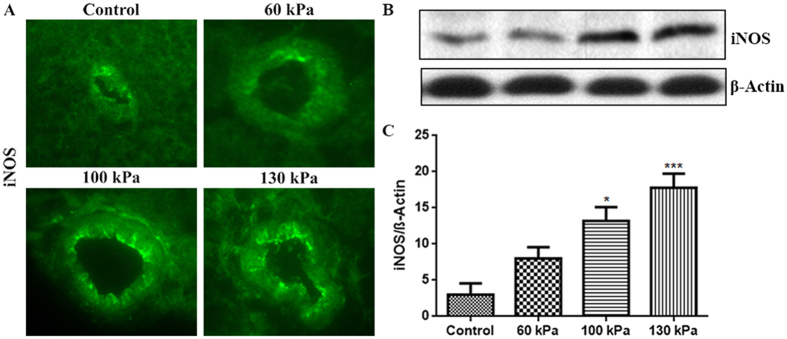
Mild TBI range of blast-wave exposure dose-dependently increased the levels of inducible nitric oxide synthase (iNOS) in rat brain microvessels. (**A**) A representative of immunofluorescent staining of iNOS in microvessel of whole brain tissue cross section in control and blast exposed animals. (**B**) Corresponding Western Blot of iNOS and housekeeping protein, β-actin. (**C**) Bar graphs show the quantitative results of the iNOS immunoreactive fluorescence intensities. Values are mean ± SEM, (n = 4), and asterisks indicate statistical significance (p-value <0.05) of blast intensity groups compared with control.

**Figure 7:**
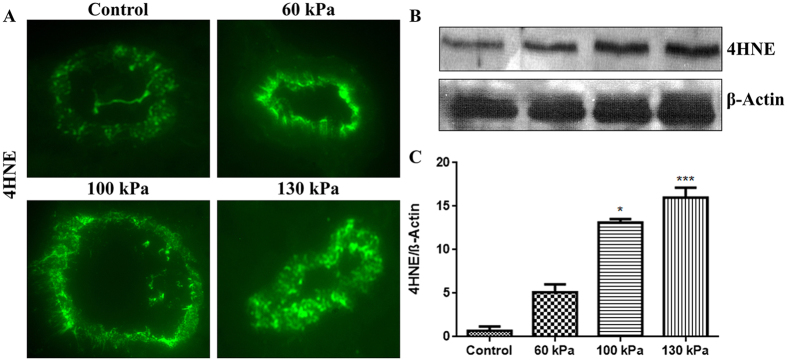
Formation of oxidative damage maker 4-hydroxynenonal (4HNE) in rat brain microvessels. (**A**) A representative of immunofluorescent staining of 4HNE in microvessel of whole brain tissue cross section in control and blast exposed animals. (**B**) Corresponding Western Blot of 4HNE and housekeeping protein, β-actin. (**C**) Bar graphs show quantification results of the 4HNE immunoreactive fluorescence intensities. Values are mean ± SEM, (n = 4), and asterisk indicates statistical significant (p-value <0.05) of each blast intensity compared with control.

**Figure 8:**
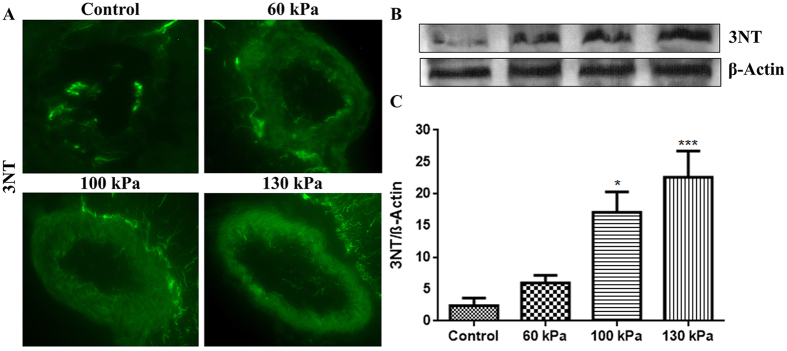
Formation of nitrosative damage maker 3-nitrotyrosine (3NT) in brain microvessels of low intensity blast range. (**A**) A representative of immunofluorescent staining of 3NT in microvessel of whole brain tissue cross section in control and blast exposed animals. (**B**) Corresponding Western Blot of 3NT and housekeeping protein, β-actin. (**C**) Bar graphs of quantification of the 3NT immunoreactive fluorescence. Values are mean ± SEM, (n = 4), and asterisk indicates statistical significant (p-value <0.05) of each blast intensity compared with control.

Using our logistic regression risk injury model, we can define the upper level of mTBI at 145 kPa BOP as 5% PMR. We examined the oxidative/nitrosative injury and the BBB integrity in the range of 60 kPa to 130 kPa peak overpressure and determined lower threshold of mTBI at 80 kPa. These markers were evaluated 24 hours after the injury, at a time point which proved in our previous study to yield their maximum levels in analogous bTBI model^50^. We first evaluated the induction of free radical generating enzymes NADPH oxidase (NOX1) and inducible nitric oxide synthase (iNOS) in the brain capillary cross section tissues. We found that blast-wave exposure significantly up-regulated the induction of NOX1 with 100 kPa (p = 0.02) and 130 kPa (p = 0.001) BOP (Fig. 5A–C). Similarly, iNOS expression was increased at 130 kPa BOP (p = 0.0005) and at 100 kPa BOP (p = 0.04) (Fig. 6A–C). Induction of NOX1 and iNOS produces superoxide and nitric oxide respectively, which will also react together to form peroxynitrite, a more reactive free radical. The oxidative/nitrosative damage is a post-oxidant production event. Proteins adducted with 4-hydroxynonenal (4HNE, oxidative stress marker) or 3-nitrotyrosine (3NT, nitrosative stress marker) are used for assessing the extent of injury in the tissue. In parallel with the induction of NOX1 and iNOS enzymes, we found that the level of oxidative damage signature 4HNE (Fig. 7A–C) is increased in mTBI range exposure with 100 kPa (p = 0.05) and 130 kPa (p = 0.0004). Similarly, 3NT (Fig. 8A–C) was found to be increased with the mTBI range exposure (100 kPa BOP, p = 0.04 and 130 kPa BOP, p = 0.002).

### Disruption of the BBB integrity

**Figure 9:**
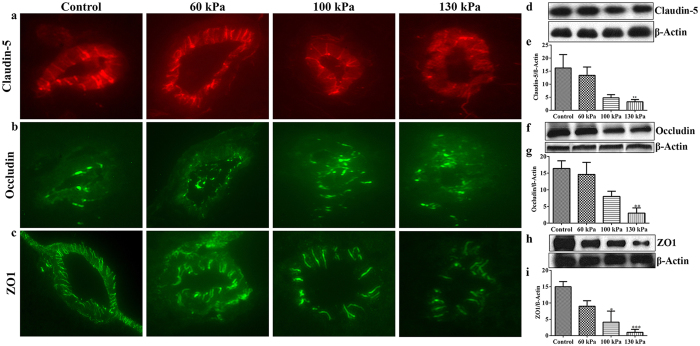
Oxidative/nitrosative damage of capillaries impaired BBB integrity by disrupting the tight junction proteins. (**a**) A representative of immunofluorescent staining of TJ protein Claudin-5 (**a**), Occludin (**b**), and zonula occluden 1 (ZO-1, **c**) in capillaries of whole brain cross-sections in control and blast exposed animals. (**b**) Corresponding Western blot and bar graphs showing the quantification of respective immunoreactive fluorescence intensity of Claudin-5 (**d**,**e**), Occludin (**f**,**g**), and zonula occluden 1 (**h**,**i**). Values are mean ± SEM, (n = 4), and asterisk indicates each blast intensity compared with control where level of statistical significance was achieved (p < 0.05).

The capillary oxidative/nitrosative damage might lead to BBB disruption, and thus we evaluated the alterations of tight junction (TJ) proteins claudin-5, occludin and zonula occluden 1 (ZO-1). TJ proteins are the primary functional barrier biomolecules of the BBB. A reduction in TJ protein levels or disruption of the architectural structure of any TJ protein is expected to impair BBB integrity, thereby enhances the chance of immune cell infiltration into the brain for initiation of neuroinflammation. Interestingly, our results showed that mTBI range of blast-wave exposure decreased the levels of claudin-5 (130 kPa, p = 0.001) (Fig. 9a,d,e), occludin (130 kPa, p = 0.002) (Fig. 9b,f,g) and ZO-1 (100 kPa, p = 0.03; 130 kPa, p = 0.0007) (Fig. 9c,h,i) proteins in brain tissue sections. These data suggest possible leakiness of the BBB and it might result in neuronal inflammation around the perivascular region of the brain.

### Assessment of BBB leakage

**Figure 10:**
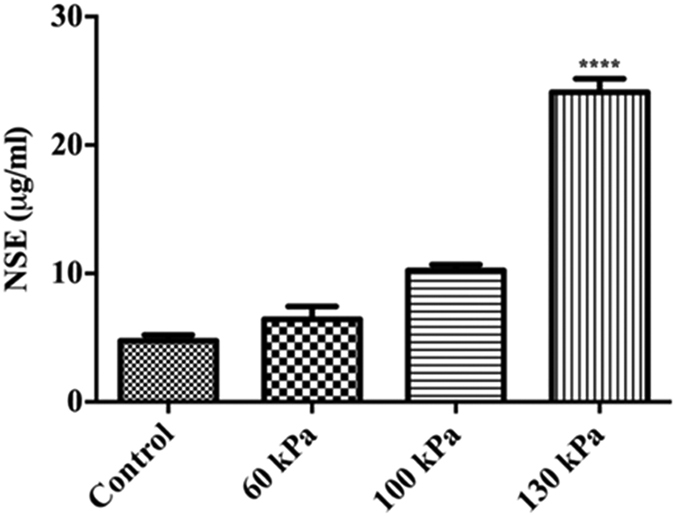
The neuronal damage marker leakage into serum is facilitated by BBB disruption. ELISA results show the levels of neuronal specific enolase (NSE) in rat blood serum collected from a single blast exposure at 60, 100 and 130 kPa. Blood samples were collected at the time of sacrifice after 24 hour post blast exposure. Values are mean ± SEM, n = 4. Statistical significance (p < 0.01) compared with control was observed at 130 kPa as the lowest blast intensity.

To assess this BBB leakiness and neuronal injury, we examined the leaking out of neuronal-specific enolase (NSE) into the blood samples of tissues exposed at BOP of 60, 100 and 130 kPa versus control animals. In agreement with a decrease in BBB integrity, we observed elevation of NSE levels in blood samples exposed to blast compared with controls (Fig. 10). The glycolytic enzyme enolase is a dimeric isoenzymes and also known as neuronspecific enolase (NSE-aa, ag and gg), as these isoenzymes were initially detected in neurons and neuronendocrine cells. However, in other pathological conditions such as small cell lung cancer and neuroblastoma, NSE exhibits a signatory value in disease detection and progression. In the absence of such pathological status or stimuli, as in our experimental setup, NSE detection in plasma samples establishes BBB leakage in primary bTBI.

## Discussion

The basic question not fully elucidated yet is the relationship between pure primary blast and bTBI and its quantification using injury predictors (BOP and impulse) and suitable set of injury markers. In this way the probabilistic TBI injury scale (mild, moderate and severe) in an animal model can be developed. In this work, we have presented the survival response of 10-week old male Sprague Dawley rats in supine position under carefully controlled pure primary shock-blast loading conditions from 0–450 kPa and durations in the range of 2 msec to 8 msec. These shock loading conditions correspond to live-fire conditions of 1 to 100 kg of C4 explosives at a stand-off distance of 2 m to 10 m distance, i.e. within practical, military relevant loading conditions^54^. The explosive strengths and the stand-off distance were selected based on the data available for IEDs and landmines (see Table 1 of ref. 48) and diagnostic criteria of military mTBI, where the inclusion threshold corresponds to overpressure of 4 psi responsible for tympanic membrane rupture^55^. However, these criteria for mTBI evaluation are not based on rigorous scientific evaluation, but conservative estimates based on pressure readings by blast gages used by soldiers in the combat zone. The development of injury criteria and corresponding interspecies scaling laws should be based on the mechanisms of injury. For instance, if the injury is caused by acceleration-deceleration type biomechanical loading, then mass and momentum scaling are appropriate. In the angular rotation injury, the scaling factor for rats is of the order of 80–120^56^,^57^, when compared to humans. In this case, rotational acceleration of approximately 600,000 rad/sec^2^ will correspond to the established concussion human criterion of 6000 rad/sec^2^. However, if predominant injury mechanism is direct transmission, then stress, strain and energy are more appropriate metrics and scaling laws based on mass are inappropriate. This approach was recently demonstrated by Radovizky and co-workers with an aid of advanced computational models to bTBI scaling law between mice, pigs and humans^58^. They used peak stresses transmitted to the brain tissue as the governing criterion and determined that humans are more vulnerable to blast by merely a factor of two, when compared to mouse. They further emphasized that ICP in the brain parenchyma is affected by the impedance of skin and skull, and is not correlated with mass of body or brain. In this work, we are mainly concerned with the primary blast that excludes any head motion and hence mass based criteria are not applicable. The blast conditions have been further validated by comparing shock pulse data around a human surrogate when exposed to live-fire and in the shock tube^59^.

Our findings indicate (Fig. 2A,B), that animals exposed to BOP below a 170 kPa threshold (and corresponding 300 Pa·s impulse) have absolute survival, although presented with signs of altered biological state compared to sham, and above 300 kPa (500 Pa·s) threshold none survived. This data is captured in the form of a sigmoidal dose-response logistic regression curve with % survival response, a function of input injury predictor variables: peak overpressure and impulse (Fig. 2). In addition, we had earlier published histopathological and extensive immuno-histochemical findings that demonstrated BBB damage and neuronal degeneration around the BBB perivascular region in primary blast exposure of a single blast with peak overpressure of 130 kPa^1^,^50^. In this work we have carefully examined the brains of animals exposed to a single blast at 130 kPa and below, using biochemical changes which proved sensitive to acute brain pathology. Thus based on the survival and other complementary data, we are postulating that a range of mild (80 kPa–145 kPa), moderate (146 kPa–220 kPa), severe (221 kPa–290 kPa) and lethal (>290 kPa) be used for injury gradation. These peak overpressure ranges have the corresponding impulse values: mild (125–250 Pa∙s), moderate (251–350 Pa∙s), severe (351–450 Pa∙s) and lethal (>451 Pa∙s). We believe that BOP cannot be a single mechanical parameter defining injury, since a sharp pressure spike with a small duration will not produce the postulated injury severity. The positive phase pressure of BOP of a Friedlander type is given by equation^60^:


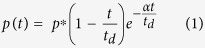


where p* is the BOP, *t*_*d*_ is the positive time duration, and α is the shape factor. I is the impulse which is the area under the pressure-time curve such that, 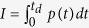 an often overlooked, but a key biomechanical loading parameter. For the same BOP, if the duration *t*_*d*_ is gradually increasing (e.g. 1 ms, 3 ms, 5 ms or 8 ms), the impulse values will follow the same trend (e.g. 40 Pa∙s, 113 Pa∙s, 183 Pa∙s, and 293 Pa∙s, respectively). Obviously higher impulse will have higher mechanical loading and hence more severe injuries, even though the BOP remains same at 140 kPa. This is analogous to the integral of acceleration and time, being used as a basis for head injury criterion in automotive accidents, or momentum (integral of force and time) in head injuries due to fall. Thus it is critical to identify both BOP and duration as two independent biomechanical parameters. Also, recent work has clearly shown that Bowen-type blast lung injury scaling laws for duration cannot be applied to blast brain injury^58^,^61^.

The injury severity classifications (mild to severe) are mostly heuristic for animal models; however, they are very important measures for inter-laboratory cross-verification of results and data interpretation. Thus such classification is necessary to advance to the next level the research on bTBI field, and facilitate development of suitable strategies for prevention, diagnostic and treatment purposes. This strategy of defining a single biomechanical parameter as predictor of injury severity has worked very well for other type of animal injury models, e.g. lateral fluid percussion injury (LPI) and closed-cortical injury (CCI) models. For example, in LPI a peak inlet pressure of 50 to 120 kPa correspond to mild, 150 to 190 kPa to that of moderate and 200 to 220 kPa to severe^62^,^63^,^64^,^65^. Similarly in CCI model, a depth of penetration of 1.5 mm corresponds to mild, 2.0 mm to moderate and 2.5 mm to severe^66^,^67^.

In this work, based on the logistic regression model (Fig. 2A,B), the upper and lower thresholds of mild TBI were found to be at 145 kPa and at 80 kPa, respectively. The upper threshold defined as 5% of PMR function is the cut-off threshold. Similar injury prediction model based on survival was developed for ferret, which was also used for scaling laws in blast injury risk curves for humans^68^. A total of 64 animals were exposed to blast with the peak overpressure in the range of 98 to 837 kPa. This model has a good predictive quality (area under ROC value of 0.77), but the McFadden R^2^ value was only 0.16 for the fit, mostly because the data points at the upper extreme of blast intensities (above 900 kPa) were not collected. Further, in this model the test specimens were placed at end of the shock tube, while in our model test specimens were placed inside the shock tube. Typically shock wave profiles at end of the shock tube are characterized by shorter duration and much smaller impulse values than shock waves experienced by specimen located inside the shock tube for the same peak overpressure exposure^1^,^35^,^48^. Hence in this ferret model of blast, the animals may have been subjected to primary and tertiary loading (e.g. due to jet winds) different from that of our primary loading only condition. Hence a direct comparison between the results on ferrets outside the shock tube and our rat data inside the shock tube may not be possible. The researchers of Lovelace Foundation developed dose-response probit mortality models for a number of species for “long” (180–400 ms) and “short” (2.1–4.6 ms) duration blast waves^61^. Animals were exposed in the side-on position against the end plate in the shock tube (“long duration” group, LDG) or in prone position on a concrete pad to the high-explosive charge overhead (“short duration” group, SDG). These models developed for rats of unknown age and species have some similarity to our studies, in spite of the differences in positioning of the animals. In particular, the overpressures for (5%, 10%, 50%, 90% and 95%) mortality rates were: 1) 165, 172, 213, 255 and 290 kPa (LDG), and 2) 193, 207, 241, 282 and 303 kPa (SDG), while in our model these are: 145, 170, 245, 320 and 340 kPa, respectively (Fig. 2A). Thus, low end of our observed mortality rates matches analogous region for the LDG, but not for SDG (approx. 50 kPa differences). However, 50% mortality overpressure matches between our model and SDG, but not for LDG (difference of 34 kPa). On the high-end of mortality curve (90% and 95%) we observe shift towards higher peak overpressures in our model compared to both, LDG and SDG by more than 40 kPa.

Riesling and co-workers^69^ reported exposure to “pressure above 236 kPa results in lethal bleeding from the airways in more than 50% of the animals”. However, there are clearly major differences between both models: while in their model rats were positioned perpendicularly to the direction of the blast and the side of the animal was exposed, in our model the animal is positioned head on and in parallel orientation with respect to incoming shock wave. We did not observe any bleeding from airways in our studies.

We observed that the thresholds for pulmonary injury and bradycardia were higher than that of brain injury^70^,^71^. The main reasons for diminished pulmonary injury could be attributed to the protective aluminum shielding in the prone position during blast exposure, characteristic for our model. The prone position with the protective aluminum may represent the protective body armor of soldiers, thus leading to the possibility that brain injury occurs at lower BOP than lung injury^72^. Soldiers are either in vertical or horizontal position while wearing body armors typically made of Kevlar-epoxy exterior with ceramic plates surrounded by rubber to contain the high velocity projectiles and shrapnel. In general, when an air shock encounters a structure, the proportion of energy reflected and transmitted will depend on the acoustic impedance mismatch between air and the structure. Impedance of a material is given by Z = ρc where c is the acoustic velocity in the medium and ρ the density. Transmission coefficient of energy is given by: 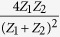, where Z_1_ and Z_2_ are the impedance of air and medium^57^. Since impedances of aluminum and body armor material are very high, transmission is negligible. This observation was also independently verified in a number of experiments performed in our laboratory using different structural materials and measuring the pressure of the transmitted shock wave. Similarly, soldiers wearing body armors have reported no thoracic injuries, still reporting mild to moderate TBI^73^. In the present study, it is clear that, lung injury cannot be linked to the cause of death even at extreme BOP range: a number of animals exposed to BOP in the range of 180 to 300 kPa (300 to 500 Pa·s) died without apparent lung injuries (injury score of zero was recorded, see Fig. 3C,D). Bradycardia, mediated by vagal reflex, is one of the frequently reported hallmark physiological responses after the blast exposure^74^,^75^,^76^,^77^,^78^, similar to our present findings. Although, we observed bradycardia even at the very low blast intensity (60 kPa), it was not statistically significant from one group to the other because the variability of results within the same discrete blast intensity were relatively big.

Finally, induction of oxidative/nitrosative damage of the BBB^30^,^33^,^50^,^79^,^80^ and subsequent impairment of the BBB is now considered one of potential mechanisms of traumatic brain injury in blast-wave exposure^30^,^33^,^50^,^51^,^52^,^79^,^80^,^81^,^82^,^83^. Here, we use disruption of the BBB integrity to determine the lower threshold of the mTBI in bTBI rat model, while the upper threshold was based on PMR curve. Thus, based on our logistic regression model the upper threshold of mTBI was set at 145 kPa, while based on oxidative or nitrosative damage of the BBB, the lower threshold of mTBI was established at 85 kPa.

In this work, we have exposed rats to a wide range of intensities of primary blast waves (below 130 kPa), where we found observable changes in acute TBI biomarkers with 100% survival. When the animals are exposed to the blast with peak overpressure higher than 300 kPa (500 Pa·s), we observed only fatalities. The pooled survival data were used to calculate the PMR (response) as a function of BOP and impulse (input). While the results clearly delineate the different TBI injury severity levels in a wide range of BOP (0 to 450 kPa), it should be recognized, that these numbers should be used with caution. These data refer to the pure primary blast wave acting on a male, 10 week-old, 320–360 gram, Sprague-Dawley rats in prone position when the body is aligned with head facing the on-coming shock wave and the thoracic region protected by metallic plate (Fig. 1B). Any change of these conditions may alter the response and hence the upper and lower thresholds BOP for a given injury severity can be different.

Thus, caution needs to be exercised in using these biomechanical parameters as universal injury predictors for variety of blast experimental setups presently used in various laboratories. Currently, five possible biomechanical loading mechanisms are identified as potentially responsible for bTBI: direct shock wave transmission to the brain through the skin/skull^59^; skull deflection induced pressure wave in the brain^84^; acceleration/deceleration loading due to pressure forces^43^; cavitation due to reflection at the back side of the skull or underpressure^85^; and pressure surge to the brain caused by loading of the thorax^31^. The shock pressure loading in the brain (exhibited as intracranial pressure or biomechanical stretching/twisting) is governed by how the external shock wave is transmitted to the brain. That in turn is determined by the thickness of the skull, which varies with the age, weight, gender and strain of the rats. Hence any variation in this factor may affect the geometry and material properties and response to shockwave and hence the effective brain biomechanical loading. Any change in biomechanical loading of the brain tissue will alter the injury severity and shift the survival curve, either to the right or left. Similarly, while in prone position the thoracic region is protected, the testing in supine position may result in increased brain injury severity caused by pressure surge, and more pronounced lung injury. Both effects could affect the outcome, shifting the PMR function towards lower BOPs. In some laboratory settings, the rats are placed in cages, tied to the end of the rod, or inserted in a tube from the side. Such constraints will alter the loading and cause differential motion of parts not immobilized, resulting in additional injuries that may affect the response, which cannot be classified as primary blast injury. While we believe that our results are reproducible in age-matched rats in the same age group under similar loading conditions, care should be taken that the experimental conditions are causing primary bTBI and not mixed or other types of injuries. These, non-primary injuries, will likely be observed if animals are kept under non-ideal exposure conditions (e.g. near the exit of the shock tube, independently whether it is inside or outside) and in smaller shock tubes (which will result in specimen overload caused by excessive reflected pressure buildup, when the animal body blocks the shockwave pathway).

Also, while we have used damage to neurovascular unit as possible markers to gauge the extent of TBI, application of other markers are equally viable. The wide variety of available imaging, electrophysiological, behavioral, cellular/molecular biomarkers can be used for this purpose and serve as response correlates. Such comprehensive approach will allow fine tuning of the threshold ranges for the various TBI injury severity levels.

## Materials and Methods

### Animals

Adult 10-weeks-old male Sprague-Dawley (Charles River Laboratories) rats weighing 320–360 g were used in all the studies. The animals were housed with free access to food and water in a 12-h dark-light cycle at 22 °C. All procedures followed the guidelines established in the Guide for the Care and Use of Laboratory Animals and were approved by the Rutgers-Newark Institutional Animal Care and Use Committee (IACUC). We used a total of 280 rats for three different experiments consisting of mortality rate in response to blast dose, evaluation of cardiac physiology, and evaluation of lung injury.

### Mortality rate in response to primary blast

Rats were exposed to a single blast wave at the Center of Injury Biomechanics, Materials and Medicine (New Jersey Institute of Technology) in the modular, multi-size shock tube capable of reproducing complex shock wave signature (Fig. 1A,B)^2^,^86^,^87^. We have evaluated a 24-hour survival of the animals following exposure at 60, 100, 130, 160, 190, 230, 250, 290, 350 and 420 kPa peak overpressure range (Fig. 1C). We have used 13 rats for each discrete incident peak overpressure exposure (13 × 10 + 10 control rats = 140 rats). These results were used to develop a logistic regression model of peak overpressure and impulse as a function of survival.

All rats were anesthetized with mixture of ketamine and xylazine (10:1 (100 mg/10 mg/kg), 0.1 mL/100 g) administered via intraperitoneal injection. Sham control rats received anesthesia and noise exposure but without blast exposure, i.e. anesthetized animals were placed next to the shock tube and then a single shot was fired. Specimen was mounted in the test section located inside of the shock tube, i.e. 2.80 m from the breech (3.05 m from the exit) for each discrete incident peak overpressure. The adjustment of Mylar membrane and keeping the breech length constant at 21.6875 inches (0.5508 m) controlled the incident pressure. We have used an aerodynamically optimized aluminum bed designed as a holder for shock wave exposure of small rodents. The riser attached to the holder helps to position the specimen in the center of the shock tube away from the walls. All rats were tested in a prone position and were strapped securely to the bed with a thin cotton cloth wrapped around the body (Fig. 1B). The strapping does not protect the animal from the intensity of the shock wave, but eliminates head motion that was verified in separate set of experiments. After experiments animals were returned to the holding room and were maintained on warming pads until recovery from anesthesia, to prevent any neuroprotection associated with hypothermia^88^.

As a quality control measure, we have also monitored a high-speed video recording to capture any substantial head/body motion during the blast. The rationale is to exclude the impact of secondary/tertiary blast injuries. The high-speed camera video recording system with a Photron FASTCAM Mini UX100 operating at framerate of 5000 fps was used and typically 2 seconds of video footage per experiment were recorded. The recorded videos are then saved via PFV (Photron FASTCAM Viewer) 3.3.5 software. The incident overpressure at the location of the animals in the test section is recorded by a custom LabView program running on in-house built data acquisition system based on National Instruments PXI-6133 32 MS Memory S Series Multifunction DAQ Modules and PXIe-1082 PXI Express Chassis. Pressure sensors used in our experiments were PCB Piezotronics (Depew, NY) model 134A24. All data were recorded at 1.0 MHz sampling frequency. Typical acquisition times were 200 ms per experiment.

### Evaluation of lung injury

Animals were anesthetized as described above and sham control rats were subjected to noise exposure. We used 10 rats per each discrete incident peak overpressure at 130, 160, 190, 230, 250, 290, 350, 390 and 420 kPa, and sham control using a total of 100 rats. Rats were sacrificed immediately after the blast exposure and the lungs were surgically removed from the thoracic cavity and the lungs were placed in 30–40 mL of freshly prepared 10% formalin solution. The severity of lung injury was performed using the Yelveton’s Pathology Scoring System^53^. The severity of injury (IS) is defined by the equation:


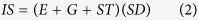


where, E is the extent of injury to the lungs (range 0–7); G is the injury grade including the surface area of the lesions (range, 0–4). ST is severity type elements, which classify the type of the worst-case lesions (range, 0–5); and SD is the severity depth element, indicating the depth or the degree of disruption of the worst-case lesion (range, 1–4). The pathological scoring ranges from zero to 64, where, 64 being the worst pathology score.

### Evaluation of cardiac physiology

For the evaluation of heart physiological function, we exposed 10 rats per discrete incident peak overpressure at 60, 100, 130, 190, 230, and 250 kPa, and sham controls following anesthetization as described. Here, we monitored the physiological vitals such as heart rate, blood oxygen saturation (spO_2_) and perfusion index that were performed at 30 minutes before and after the blast injury. The multiple vital signs were measured using MouseSTAT system (Kent Scientific Corp., Torrington, CT). Animals under anesthesia were placed on a warm pad in a supine position to prevent hypothermia. The heart rate, blood oxygen saturation (spO_2_) and perfusion index were recorded simultaneously using in-house developed LabView-based software using a pulse oximeter sensor attached to one of back paw of the rat. A rectal probe operating at a rate of 1.0 Hz was used to monitor the body temperature for 30 minutes before and after the blast exposure.

### Reagents

Antibodies from: 1) rabbit selective to: anti-NOX1, anti-iNOS, anti-4HNE, anti-Claudin-5, anti-Aquaporin-4; 2) mouse and selective to anti-3NT; 3) sheep and selective to aquaporin-4 (AQP-4) were purchased from Abcam (Cambridge, MA). Mouse antibody against Occludin was purchased from Invtrogen (Carlsbad, CA, USA). Rabbit anti-zonula occluden-1 (ZO-1) was from US Biological (Massachusetts, MA) and mouse anti-β-actin was purchased from Millipore (Billerica, MA). All secondary Alexa Fluor conjugated antibodies were purchased from Invitrogen. The ELISA kit for Neuron-Specific Enolase (NSE) was acquired from Alpha Diagnostic (San Antonio, Texas, USA).

### Immunofluorescence and microscopy

The desired post-BOP exposure and control, animals were sacrificed 24 hours after blast exposure, and freshly dissected brain tissue were embedded in OCT (Optimal Cutting Temperature) media. These preparations were stored frozen at −80 °C until ready for sectioning. Brain sections (8 μm thick) were prepared from the frozen tissue blocks, using Leica CM3050 cryostat. Tissue sections containing the external and internal capillaries were used for immunofluorescence staining. Briefly, tissue sections mounted on glass slides were washed with 10 mM phosphate buffered saline (PBS), fixed in ice-cold acetone-methanol (1:1 *v/v*) solution for 10 minutes at −20 °C. The tissue sections were blocked with 3% Bovine serum albumin (BSA) at room temperature for 1 hour in the presence of 0.4% Triton X-100. Fixed tissues were incubated overnight at 4 °C with respective primary antibodies (NOX1, iNOS, AQP-4, occludin, claudin-5 and ZO-1) containing 1% BSA and 0.3% Triton™ X-100. After washing with PBS, tissue slides were incubated with corresponding Alexa Fluor conjugated secondary antibodies for 1 hour and mounted with immunomount containing DAPI (Invitrogen). Fluorescence images were captured using fluorescent microscope Eclipse TE2000-U (Nikon, Melville, NY) with NIS elements software. Fluorescence was quantified by SigmaScan Pro program, Image Analysis version 5.0.0, 1987–1999 SPSS Inc. The fluorescence intensity quantification was carried out as the difference between the final fluorescence emitted by the tissue sections in the presence/absence of the blast exposure with reference to the background fluorescence of the tissue sections in triplicate.

### Western blotting

Cortical brain tissues were lysed with CellLytic-M (Sigma) for 30 min at 4^ ^°C, centrifuged at 14,000× g. The protein concentration in homogenate was estimated by bicinchoninic acid (BCA) method (Thermo Scientific, Rockford, IL). Subsequently, 20 μg of protein per lane was loaded into 4–15% SDS-PAGE gradient gels (Thermo Scientific). Proteins separated according to their molecular size were then transferred onto nitrocellulose membranes, blocked with SuperblockT20 (Thermo Scientific), and incubated overnight with respective primary antibody at 4 °C. Incubation with horse-radish peroxidase conjugated secondary antibodies for 1 hour, was followed by detection of immunoreactive bands by West Pico chemiluminescence substrate (Thermo Scientific). For densitometric quantitation of western blots, images were digitized using a BioRad GS800 calibrated densitometer, and analyzed with BioRad Quantity One software.

### Enzyme-linked immunosorbent assay (ELISA)

To determine the cerebral vascular BBB damage and neuronal damage by shock wave, we have analyzed blood serum samples from control and animals exposed to the blast with 60–130 kPa peak overpressure for the presence of human neuron-specific enolase (NSE/gamma enolase). The experiments were performed in triplicate with control positive peptide for NSE protein and non-immune rabbit IgG as negative control in accordance to the manufacturer’s instructions (NSE/gamma enolase kit form Alpha Diagnostic, San Antonio, Texas, USA).

### Data analysis

Statistical analysis on immunofluorescence and Western blot data was performed using one-way ANOVA, using SPSS version 22.0, and p < 0.05 were considered statistically significant. Boxplot analysis was performed to assess measurement outliers and Shapiro-Wilk and Levene’s test were performed to assess normality of data distribution and homogeneity of variances, respectively.

The logistic regression models were developed and evaluated using Systat 13.0 software (Systat Software, Inc., San Jose, CA). Dose-response models for heart rate and pulmonary injury were fitted with Origin 9.0 software (OriginLab Corp., Northampton, MA) using dose-response function:


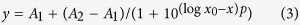


where *A*_*1*_, *A*_*2*_ are asymptotes, *log x*_*0*_ is an inflection point, and *p* is a slope value.

Power analysis was performed using freely available GPower software, version 3.1.9^89^.

## Additional Information

**How to cite this article**: Mishra, V. *et al.* Primary blast causes mild, moderate, severe and lethal TBI with increasing blast overpressures: Experimental rat injury model. *Sci. Rep.* **6**, 26992; doi: 10.1038/srep26992 (2016).
